# Vanillin decorated chitosan as electrode material for sustainable energy storage†

**DOI:** 10.1039/c9ra00140a

**Published:** 2019-02-06

**Authors:** Ivan K. Ilic, Maren Meurer, Saowaluk Chaleawlert-umpon, Markus Antonietti, Clemens Liedel

**Affiliations:** Department of Colloid Chemistry, Max Planck Institute of Colloids and Interfaces, Research Campus Golm 14476 Potsdam Germany Clemens.Liedel@mpikg.mpg.de; National Nanotechnology Center, National Science and Technology Development Agency Thailand Science Park Pathumtani 12120 Thailand

## Abstract

Energy storage materials made from bioresources are crucial to fulfil the need for truly sustainable energy storage. In this work, vanillin, being a lignin-derived molecule, is coupled to chitosan, a biobased polymer backbone, and used as a redox active electrode material. The structure of those electrodes is highly defined, leading to better product security than in lignin based electrodes, which have been presented as sustainable electrodes in the past. With over 60% of saccharide units in chitosan functionalised by vanillin, the concentration of redox functionalities in the copolymer is significantly higher than in lignin materials. Composites with carbon black require no further binders or additives to be used as electrode material and show reversible charge storage up to 80 mA h g^−1^ (respective to the total electrode material) and good stability. Consequently, these electrodes are amongst the best performing electrodes made from regrown organic matter.

## Abstract 2

To replace dangerous and rare components in battery electrodes, more sustainable energy storage materials made from biowaste and wood-based vanillin are presented.

## Graphical abstract

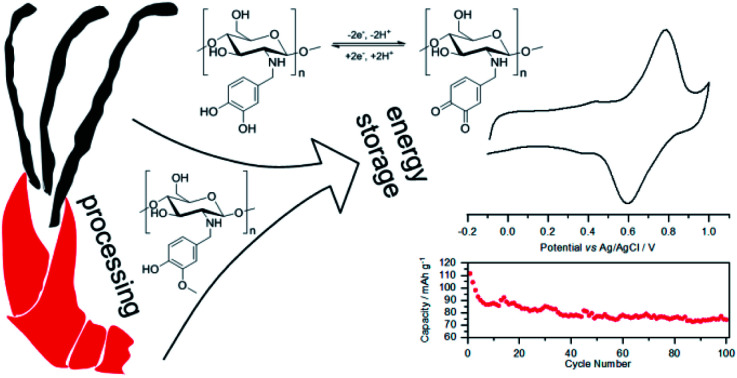

## Introduction

Endeavours to minimise humankind's impact on the environment have led to an increased use of electrical energy, which can be nowadays readily produced using renewable sources such as sun, wind, or hydropower. For example, electrical cars are popular as an alternative to fuel-driven traditional cars. For a truly sustainable society, also rechargeable batteries, becoming of increasing importance, have to be made in a sustainable way, and the standard technologies are indeed far from that point. Currently cathodes for lithium-ion batteries are mostly made from cobalt compounds, with cobalt being expensive, rare, and mined in countries being politically and socially unstable. Furthermore, losing stray batteries in the recycling loop creates hazardous waste. Therefore, there is – cost-wise and by means of the socio-ecological balance – a need for alternative cathode materials, such as organic materials made from low-value agricultural side products.^1^

**Scheme 1:**
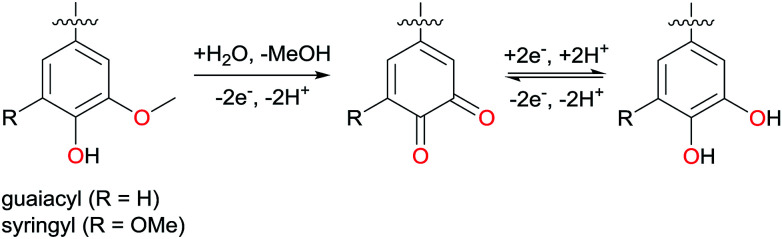
Demethylation of syringyl and guaiacyl units and the enabled quinone–hydroquinone reversible redox pair.

Lignin is a polymer that satisfies all criteria of sustainability analysis. It can be isolated from lignocellulosic biomass, but also constitutes low value side products of paper and bioethanol industry. Being the most abundant aromatic polymer on earth and with a regrowth rate of about 25 Gt per year, lignin is a promising low-cost biobased alternative to crude oil in the production of aromatic building blocks.^2^ Its complex polyphenolic structure depends, amongst others, on the plant source and the applied extraction method. All lignins however have in common that they contain guaiacyl and syringyl groups, which are electrochemically active. Consequently, lignin has already been described as a cathode material in organic batteries,^3,4^ usually after electrochemical demethylation of methoxy functionalities in those building blocks (Scheme 1).^5^ Furthermore, lignin has been described as a promising candidate for binder-free electrodes,^6^ along with other polyphenolic polymers.^7^

Vanillin and syringaldehyde, aromatic aldehydes that bear guaiacyl and syringyl groups respectively, can be readily produced by oxidation of the aliphatic fragments of lignin.^8^ Those also naturally occurring molecules are widespread in food and beverage industries, *e.g.*, vanillin is a main ingredient of vanilla flavour, while syringaldehyde gives whiskies a smoky aroma. Like other aldehydes, those molecules react with primary amines to imines. This reaction is reversible in the presence of water, but the imines can be reduced to form stable secondary amines. Therefore by attaching vanillin or syringaldehyde to an amine containing biopolymer, a sustainable redox active polymer with the functionalities of lignin is expected. The density of redox active groups depends on the density of primary amines in the biopolymer and is potentially more defined and higher than in lignin. In this regard, chitin, the second most abundant polysaccharide on earth, is especially appealing. It is an amide-rich polysaccharide that can be found in the exoskeleton and internal structures of invertebrates, but also for example in some fungi.^9^ In large scale it is obtained from biowaste, *e.g.*, crab shells. The period acetamide groups can largely be deacetylated in concentrated sodium hydroxide, and chitosan, a primary amine-containing polymer, is obtained.^9^ Being a sustainable polymer with rare primary amines, it has found already many applications such as water purification,^10^ sensing,^11^ and drug delivery.^12^

There are many reports on different chitosan based imines,^13–16^ with a special attention being given to chitosan–vanillin imines due to the ease of their synthesis that requires no hazardous chemicals. In order to create stable materials, a mild, selective reducing agent for imines (NaBH_3_CN) can be used, and series of chitosan-based secondary amines have been prepared.^17,18^ Sustainable chitosan–vanillin amines (ChiVan) have been presented for a wide range of applications such as in antibacterial devices,^19^ for encapsulation,^20^ or for heavy metal removal.^21^ Energy applications however have not been reported to the best of our knowledge.

The scope of this paper is to prepare chitosan–vanillin secondary amines and to investigate their potential to be applied as cathode materials for electrochemical energy storage. It will be shown that they possess a remarkable charge storage capacity while still remaining sustainable as their ingredients can be derived from a combination of biowaste and low value biomolecules.

## Experimental section

### Synthesis of ChiVan and ChiVan–CB

Synthesis of ChiVan was performed *via* a modified literature procedure.^17,21^ In short, chitosan (0.5 g, corresponding to 2.51 mmol of primary amine functionalities given that 85% of saccharide units in chitosan are deacetylated) was dissolved in 0.2 M acetic acid (35 mL) and stirred for 2 h. A solution of vanillin (0.48 g or 3.16 mmol, approx. 1.25 eq., in 35 mL ethanol) was then added to the mixture. After 1 h of additional stirring at room temperature, NaBH_3_CN (0.77 g, 12.22 mmol, 4.9 eq.) was added and the reaction stirred further for 17 h. A precipitate formed and was dissolved by addition of glacial acetic acid (3 mL). For ChiVan–CB, carbon black (1.5 g), that was previously grinded in an agate mortar, was added after 1 h of further stirring and stirred for 24 h. The mixture was neutralised using a 15% aqueous solution of Na_2_CO_3_ and stirred for 2 hours. The product was centrifuged by splitting the dispersion in two centrifugation tubes (max capacity 50 mL) and subsequently washed two times with water and two times with ethanol (each centrifugation was done at 4000 rpm for 3 minutes). The obtained solid was dried at 60 °C overnight. Syntheses with other ChiVan-to-carbon black ratios were done accordingly, and they are all described in detail in the ESI.†

### Materials and instrumentation

Chitosan with an average molecular weight of 15 kDa and with minimum 85% degree of deacetylation (Polysciences, Inc.), vanillin (Sigma-Aldrich), syringaldehyde (Acros Organics), 4-hydroxybenzaldehyde (Sigma-Aldrich), sodium cyanoborohydride (Acros Organics), sodium carbonate (Carl Roth), kraft lignin from southern pine trees (Domtar), carbon black AB-520 (MTI Corporation), and carbon paper Spectracarb 2050A-0550 (Fuel Cell Store) were used as received. Solvents acetic acid (VWR International), ethanol (Fisher), propylene glycol (Acros Organics), and deuterated solvents water (Sigma-Aldrich) and acetic acid (Sigma-Aldrich) were used without further purification. The surface morphology of carbon black, ChiVan–CB composite and the composite after grinding was investigated by SEM using a Zeiss Leo Gemini 1550 microscope. Nitrogen adsorption measurements were performed using a Quantachrome Quadrasorb SI porosimeter with N2 at 77 K. ^1^H NMR samples were prepared by dissolving the material (10 mg) in D_2_O (1 mL) and D_3_CCOOD (10 μL). The spectra were measured using an Ascend 400 MHz NMR spectrometer (Bruker). FT-IR measurements were performed at a Nicolet iS 5 FT-IR Spectrometer (ThermoFisher Scientific). ICP-OES was conducted using a Horiba Ultra 2 instrument equipped with photomultiplier tube detection. Samples were dissolved in aqua regia and filtered prior to analysis.

### Electrochemical measurements

All the electrochemical measurements were performed in the same setup (Fig. S2†). 1 M HClO_4_ (50 mL) was used as an electrolyte with a platinum wire counter electrode and an Ag/AgCl reference electrode in saturated KCl solution. For the working electrode, different composites (60 mg) were grinded using a planetary ball mill PM100 (Retsch) equipped with stainless steel jars and balls for 50 min and for another 10 min together with 250 μL propylene glycol. Ethanol (750 μL) was added to the slurry, and samples were hand shaken and ultrasonificated for 5 min. 40 μL of the obtained slurry was spread across a carbon paper (1.5 cm^2^). For electrodes with higher or lower loading, the volume of slurry to be spread on carbon paper was adjusted accordingly.

## Results and discussion

**Scheme 2:**
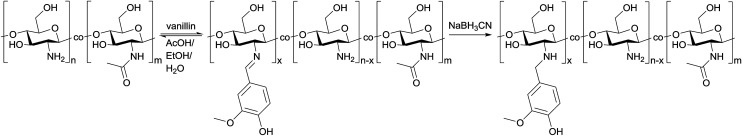
Formation of chitosan–vanillin graft copolymer; *n* ≈ 85%, *m* ≈ 15%, and *x* ≈ 62%.

Synthesis of the chitosan–vanillin graft copolymer (ChiVan) was performed according to a modified literature procedure (Scheme 2).^17,21^ Chitosan, which naturally contains primary amines as well as acetamide groups due to incomplete deacetylation of chitin, was firstly dissolved in 0.02 M acetic acid due to the good solubility of chitosan in aqueous diluted solution of weak organic acids. Secondly vanillin was added, leading to the formation of imine bonds between the primary amines and vanillin in a slow reversible reaction. The imines were irreversibly and selectively reduced to secondary amines upon addition of sodium cyanoborohydride. While stirring overnight, a white precipitate formed due to the rise of pH as a consequence of imine reduction that consumes protons.^17,21^ The redissolved polymer by acidification was purified and characterised using nuclear magnetic resonance (NMR) and Fourier-transform infrared (FT-IR) spectroscopy.

**Fig. 1:**
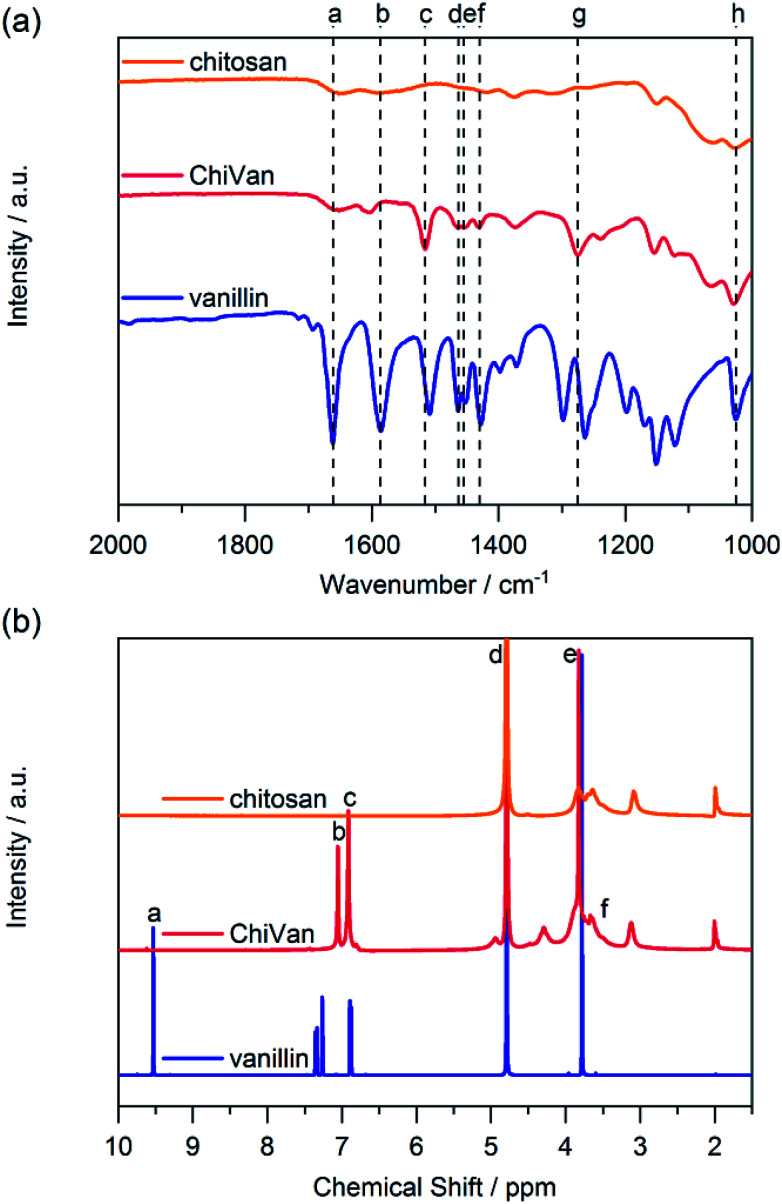
(a) FT-IR and (b) ^1^H NMR spectra of chitosan, ChiVan, and vanillin in 1% solution of deuterated acetic acid in deuterated water.

We performed FT-IR measurements on chitosan, ChiVan, and vanillin (Fig. 1a). In vanillin, the C=O stretching vibration can be observed clearly at 1661 cm^−1^ (a), while this band does not appear in the spectrum of ChiVan, confirming the reaction of the aldehyde group in vanillin and successful removal of any excess vanillin. A weak band in the spectra of chitosan and ChiVan at a similar position can be ascribed to the C=O stretching vibrations of acetylated groups in chitosan (*cf.*Scheme 2). Also the band at 1586 cm^−1^ (b) in the spectrum of vanillin, which represents a conjugated interaction between the aromatic ring and the carbonyl group, does not appear in the spectrum of ChiVan. The absence of any other band in ChiVan between 1600 and 1700 cm^−1^ points to the successful reduction of imine bonds in ChiVan. Proofs that the vanillin functionalities were successfully incorporated in ChiVan can be seen at 1516 cm^−1^ (c, aromatic C–C vibrations), 1463 cm^−1^ and 1430 cm^−1^ (d and f, asymmetrical CH_3_ deformation vibrations), 1455 cm^−1^ (e, symmetrical CH_3_ deformation vibrations), 1275 cm^−1^ (g, rocking vibrations for CH_3_ groups), and 1025 cm^−1^ (h, mode of O–CH_3_ group vibration).^22^

Furthermore, we investigated ChiVan by ^1^H NMR spectroscopy and compared its spectrum to the ones of chitosan and vanillin (Fig. 1b). The sharp peak at 4.79 ppm (d) corresponds to deuterated water.^23^ No free vanillin is left inside ChiVan as confirmed by the disappearance of the aldehyde proton peak at 9.53 ppm (a). Peaks at 7.05 and 6.91 ppm (b and c, aromatic protons) and at 3.82 ppm (e, methoxy proton) as well as peaks in the range of 3–4 ppm (f, protons at the saccharide rings) indicate successful formation of a polymer, in which vanillin-derived guaiacyl groups are grafted onto a chitosan backbone. NMR spectroscopy further proves the successful reduction of imines (*cf.*Scheme 2) as no signals at higher chemical shift than the aromatic proton peaks, where imine protons in chitosan–vanillin imines would be expected, can be detected in ChiVan.

By comparing the integral of the aromatic proton peaks to the anomeric proton peak at 4.29 ppm (see ESI†), functionalisation of 62% of saccharide rings by guaiacyl groups is calculated which agrees well with previous literature.^21^ The remaining 38% of saccharide rings do not contain grafted vanillin units but either acetamide functionalities (up to 15% according to the vendor of chitosan) or unreacted primary amines. Given that 62% of saccharide units are functionalized with vanillin derived guaiacyl groups in the final polymer, the concentration of free guaiacyl groups in the bioderived polymer is 2.46 mmol g^−1^, which is significantly higher than in common lignins.^24^

ChiVan is a non-conductive material. For using ChiVan as a cathode material, we prepared a composite with carbon black to enable charge transport. The method of electrode preparation can significantly alter the properties of an electrode. As a method of choice, the composite of carbon black and ChiVan (ChiVan–CB) was grinded in a ball mill with propylene glycol and ethanol as a sustainable dispersion agent.^25^ Details of the experimental procedures are summarized in the Experimental part. No binder was used in the electrode preparation as ChiVan acts not only as an active material but also as a binder, similar to chitosan.^26^ Omission of the binder simplifies the cathode material and increases its sustainability as molecules like the most commonly used polyvinylidene difluoride (PVDF) are avoided.

**Fig. 2:**
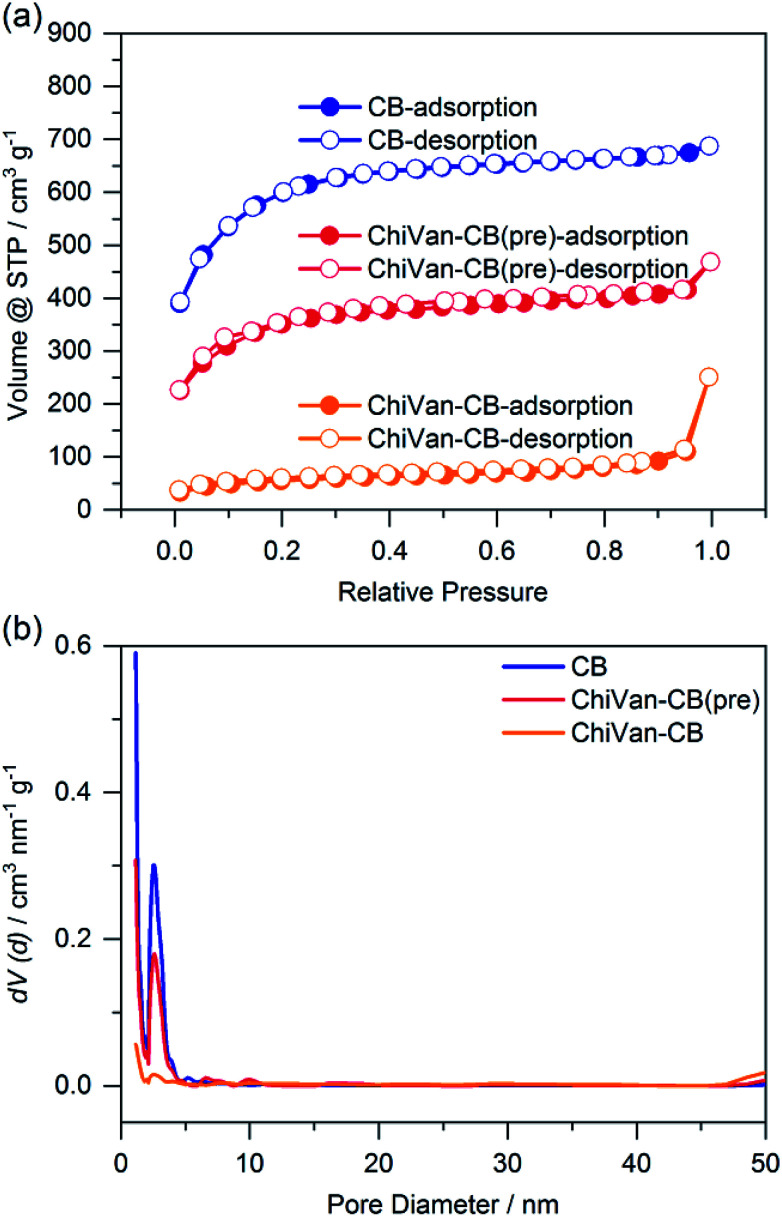
Nitrogen sorption measurement of carbon black (blue), a ChiVan–CB mixture (red), and ChiVan–CB after grinding (orange). (a) Isotherms and (b) pore size distribution.

For analysis, the prepared ink was dispersed in a large excess of ethanol. After centrifugation, the solid was dried at 80 °C overnight and investigated without further modifications. This procedure was done in order to remove remnants of propylene glycol which is hard to completely remove in vacuum due to its high boiling point. Thermogravimetric analysis (TGA) investigations in Fig. S4 (ESI†) confirm the formation of a ChiVan–CB composite, as its thermogravimetric behaviour resembles a combination of ChiVan and carbon black. Fig. 2 shows the nitrogen sorption behaviour of carbon black and of the composite material before and after ball milling.

As can be observed in Fig. 2 the apparent specific surface of carbon black (2117 m^2^ g^−1^) drops after the introduction of ChiVan (1250 m^2^ g^−1^), most likely because of lower specific portion of porous material, and it drops even further after grinding (190 m^2^ g^−1^). Furthermore, carbon black is microporous with a significant amount of mesopores (diameter of 4 nm). After the introduction of ChiVan and even more significantly after ball milling, the apparent pore volume of micro- and mesopores decreases, while there are apparently more macropores. Obviously, the surface area available for nitrogen adsorption decreases in the course of the process, which may be due to remaining solvent with a high boiling point in the pores, adhesion of ChiVan to the surface of the carbon black, gluing of the pores, or partly filling of the pores with ChiVan. The high pressure created by the impact with the ball during the grinding process may also influence the apparent surface area, for example by breaking the carbon black particles and increasing the surface area between individual small particles, which then appears as macropores. ChiVan binds different grains of carbon black, making them immobile and creating macropores between them.

**Fig. 3:**
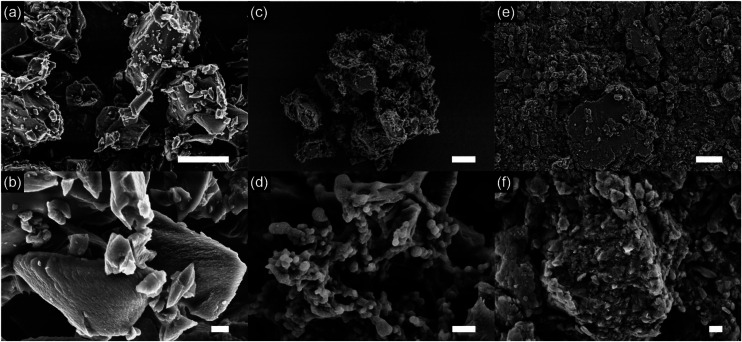
SEM images of carbon black (a and b) and ChiVan–CB before (c and d) and after (e and f) grinding. The scale bars represent 6 μm (a, c, and e) and 600 nm (b, d, and f), respectively.

In order to gain more insight to the structure, we investigated the materials by means of scanning electron microscopy (SEM). Fig. 3 shows the results.

In all samples, carbon black particles can be observed in the range between few hundred to few thousands of nanometres, and energy dispersive X-ray spectroscopy (EDX) measurements detect only carbon, oxygen and small amounts of nitrogen (Fig. S5†). Upon addition of ChiVan, EDX measurements detect significant amounts of nitrogen that is concentrated to the regions of polymeric structure, a proof of ChiVan–CB composite formation. After ball milling, significantly smaller particles in the range of tens to hundreds of nanometres can be detected. No free polymer structures are observable, which may confirm a good interaction of carbon black and ChiVan as indicated by nitrogen adsorption measurements. Furthermore, ball milling homogenises ChiVan, as can be seen by studying nitrogen and oxygen content, which are uniform through the sample and significantly higher than in the case of carbon black. ChiVan acting as binder is supposed to hold different carbon particles together. Such a tight composite should allow for an easy transport of electrons between carbon black and ChiVan, making it a preferred material to be used in batteries.

We note that ball milling using iron jars and balls may incorporate iron particles into the final material. Consequently, we investigated iron contamination using inductively coupled plasma – optical emission spectrometry (ICP-OES). ChiVan–CB has 0.040 (std. dev. 0.0002) mg g^−1^ of iron in it, while after grinding it has 1.54 (std. dev. 0.023) mg g^−1^. These low levels of contamination are expected due to the softness of milled material,^27,28^ however, they are assumed to be too low to have any significant influence on the electrochemical energy storage properties of our materials.

**Fig. 4:**
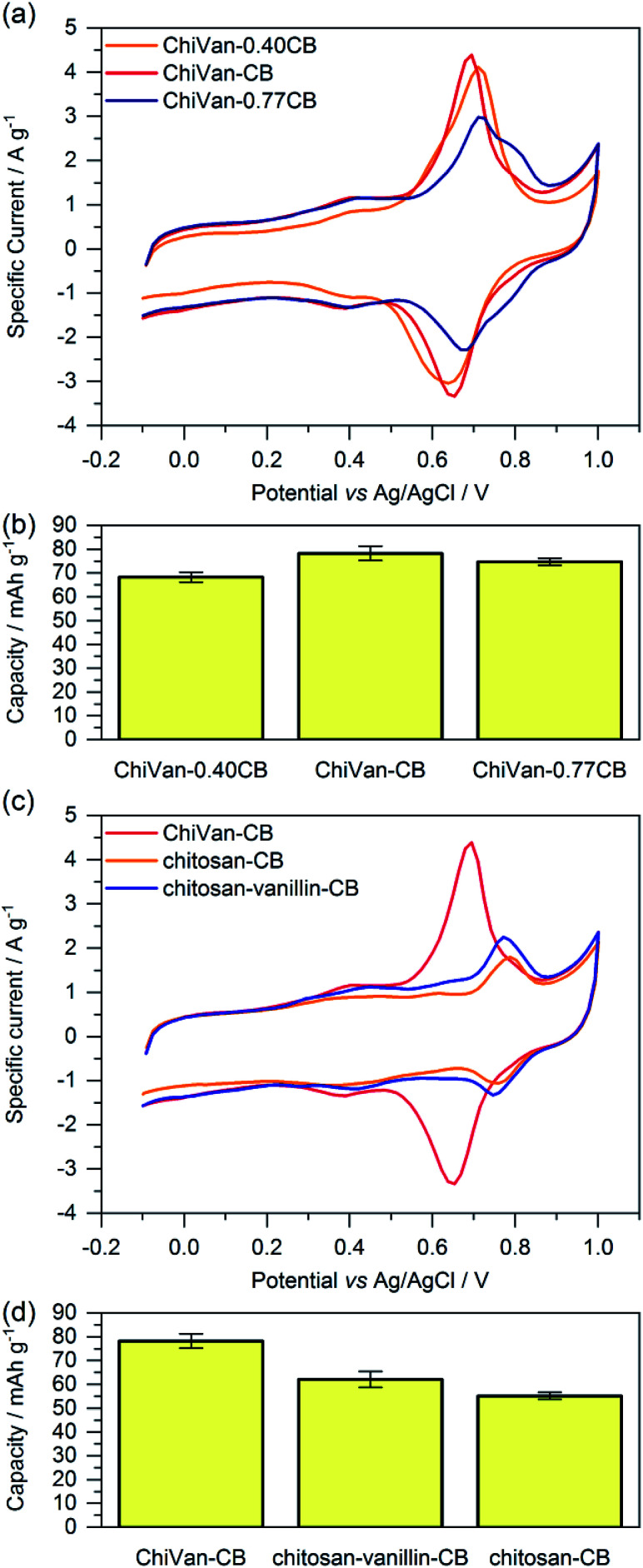
(a and c) Cyclic voltammetry and (b and d) average capacities as calculated by integration of the cyclic voltammetry curves of ChiVan–carbon black composites prepared with different ratios of both constituents (a and b) or composites of carbon black with ChiVan or other polymers in which certain steps of the ChiVan synthesis were omitted (c and d). All CV was tested in 1 M HClO_4_ aqueous solution (5 mV s^−1^). The fifth cycle of the respective experiment is displayed.

For investigating the electrochemical performance of the composite, the prepared ink was spread over graphite paper after ball milling. No washing with ethanol was performed as we do not expect possible traces of remaining propylene glycol to negatively influence charge storage performance. Fig. 4 shows cyclic voltammograms and capacity as calculated by integration of the cyclic voltammetry (CV) curves of different electrode compositions. It is important to note that ChiVan, just like lignin, undergoes electrochemical demethylation during the first oxidation step.^5^

Composites of ChiVan and carbon black with different ratios of the constituents (40%, 67% and 77% of carbon black) are used to determine the optimal ratio between ChiVan and carbon black. Fig. 4a and b summarises cyclic voltammograms (fifth cycle) and capacity as calculated by integration of CV curves of different compositions. All three composites show a rectangular background, characteristic for porous carbons due to the formation of an electric double layer, and a pronounced pair of redox peaks between 0.6 and 0.7 V *vs.* Ag/AgCl, characteristic for polymers that undergo reversible *o*-quinone to *o*-hydroquinone transformation. Specific currents and specific capacities in the figure and throughout this manuscript all refer to the mass of total composite electrode material including carbon black and not to the mass of active material alone as the shape of CV curves indicates a significant contribution of carbon black to charge storage. The composite ChiVan–CB (approximately 67% CB in the composite electrode) shows the highest specific capacity, visible in cyclic voltammetry both in terms of the highest redox couple peak and the highest rectangular background, meaning that it has the optimal ratio of ChiVan to carbon black. ChiVan–0.77CB (approximately 77% CB in the composite electrode) has too much carbon black in it, so the redox couple constitutes a lower proportion of the total mass, resulting in a less pronounced redox peak in CV curves and a lower specific capacity (calculated respective to the total mass of electrode material). The relatively increased amount of carbon black does not lead to a more pronounced rectangular background in CV curves representing non-faradaic charge storage, probably due to limited accessibility to the electrolyte. ChiVan–0.4CB (approximately 40% CB in the composite electrode) in contrast has too much ChiVan in it, leading to insufficient electron transport. As a result of lower relative amount of carbon black, the rectangular background in CV curves is less pronounced, leading to lower capacity. Therefore, all further mentioned composites were made using approximately 67% CB during synthesis (33% ChiVan or other polymer in the composite electrode). We emphasize that both constituents contribute to charge storage and all specific capacities are calculated referring to the total mass of the electrode, not to the mass of ChiVan.

In order to asset the contribution of vanillin in ChiVan to charge storage and to investigate the necessity of imine reduction during synthesis, composites of the organic material and carbon black were prepared, however without the addition of vanillin (chitosan–CB) or reduction step (chitosan–vanillin–CB) during synthesis, respectively (Fig. 4c and d). Composites without chitosan, which would be interesting to investigate the influence of the binding polymer backbone, could not be fabricated. When omitting addition of chitosan during synthesis, the resulting dispersion did not form a processible slurry, illustrating the necessity of chitosan due to its binding properties. In CV measurements of samples without vanillin, no redox couple between 0.6 and 0.7 V can be observed. This observation additionally proves that the redox pair in this range corresponds to the vanillin moiety in ChiVan. Samples which were synthesised without reducing the imine groups (*cf.*Scheme 2) also do not show a redox couple in this region, probably due to the hydrolysis of imine bonds in the acidic electrolyte. These results demonstrate the versatility of chitosan in ChiVan, on one hand as a binder of carbon black,^26^ and on the other hand to prevent vanillin dissolution in the electrolyte.

We note that polymers in which vanillin was replaced by another aldehyde which bears functional groups of lignin (syringaldehyde or *p*-hydroxybenzaldehyde) have a lower specific capacity and lower redox potential of the quinone functionality (if present). Similarly, replacing ChiVan with Kraft lignin results in a lower specific capacity and lower redox potential. Details are summarized in the ESI.†

**Fig. 5:**
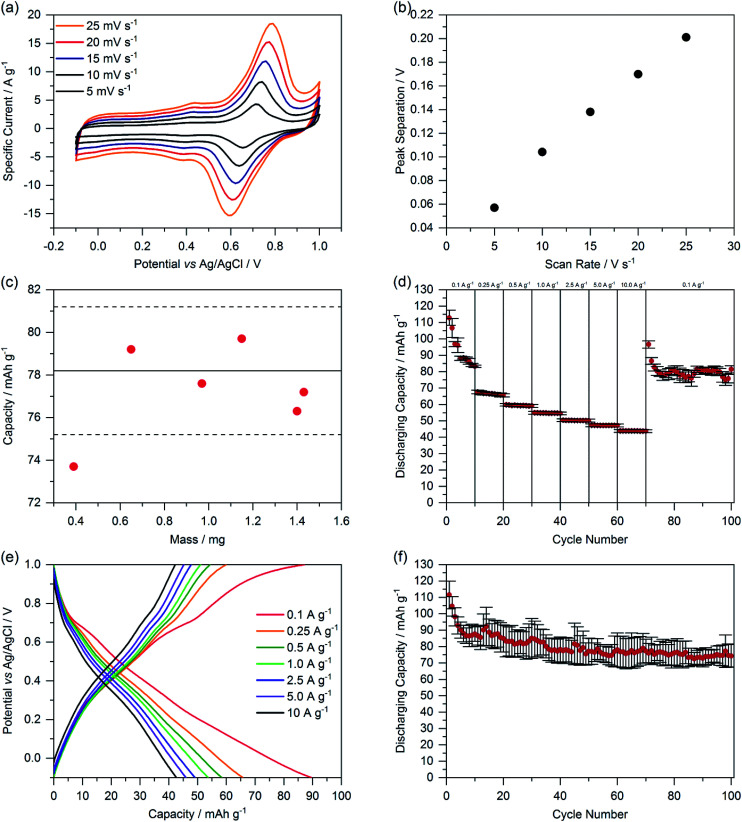
(a) Cyclic voltammetry of ChiVan–CB with different scan rates. (b) Oxidation and reduction peak separation depending on the scan rate. (c) Capacity as calculated by integration of CV curves with different loadings on the working electrode. The full and dashed lines represent the average capacity and standard deviation of the capacity of ChiVan–CB electrodes described above in Fig. 4. (d) Rate performance at different current densities. (e) Galvanostatic charging–discharging measurements. (f) Rate performance for 100 cycles at a current density of 0.1 A g^−1^. All the electrodes were prepared by mixing 33% of ChiVan and 67% carbon black. The prepared electrodes were used as working electrodes, while platinum wire was used as a counter electrode, Ag/AgCl in saturated KCl was used as a reference electrode, and 1 M HClO_4_ was used as electrolyte.

We determined the reversibility of redox reactions by measuring the peak separation of the two highest peaks in dependence of the scan rate (Fig. 5a and b). A linear dependence indicates a pseudoreversible redox reaction.^29^ Additionally, we investigated the thickness-dependency of the capacity of ChiVan–CB electrodes by adding different amounts of slurry (10–60 μL) to the current collector, resulting in different loading of composite material. The resulting capacities as calculated by integration of CV curves are summarised in Fig. 5c. All the results, except the one with the lowest loading, fall in the limits of the standard deviation of the capacity of previous measurements (Fig. 4) with constant loading of 40 μL slurry. Electrodes featuring the lowest loading have significantly lower capacity probably due to the low amount of slurry that was put on the electrode (10 μL), not allowing to be spread well over the whole surface of the electrode. These results emphasise that in our case the mass loading does not significantly affect the capacity of the electrode, probably due to the porosity of the active material. Porosity allows for the electrolyte to travel through the material, and therefore charge transfer can occur evenly throughout the electrode and not just on its outer surface.

The performance of the ChiVan–CB electrodes was finally tested in galvanostatic charging–discharging measurements (Fig. 5d–f). A nonlinear plot of potential as a function of capacity points to mixed battery-like charge storage along with capacitive charge storage, as expected for mixtures of an *o*-quinone containing polymer and high surface active carbon. Significantly pronounced capacity in the range of 0.6–0.8 V *vs.* Ag/AgCl, corresponding to the reduction peak in CV measurements, is however only observable at slow discharging rate of 0.1 A g^−1^. At higher discharging rate, the galvanostatic profile looks almost capacitor-like. The reason may be that charge transfer between quinone units and carbon black is kinetically hindered, possibly because of insufficient contact. Indeed, as visible in Fig. 5d, capacity of ChiVan–CB electrodes at high discharging rate is almost independent on the rate, indicating a surface confined charge storage behaviour. Fig. 5d and f further show that capacity of the electrode falls rapidly in the first few cycles but then is constant for 100 charging–discharging cycles, a phenomenon probably not connected with the low current density (0.1 A g^−1^), but rather due to some irreversible reactions in the first cycles. Such reactions may include crosslinking reactions between oxidized vanillin units and partly decomposition of the chitosan backbone in the strongly acidic electrolyte. In batteries, *e.g.*, lithium ion batteries that incorporate ChiVan–CB electrodes, this decomposition is not expected due to different acidity of the electrolytes. Such experiments however are beyond the scope of this study and will be investigated in the future.

## Conclusions

To summarise, organic electrode materials based on low value biomass were successfully synthesised using only sustainable chemicals. The active polymer material was chitosan-derived, which is available from crab shell waste, with grafted vanillin units, deriving from lignin and as such from a low-value side product of paper industry. The polymer was characterized and composited with carbon black to form a tight composite. Nitrogen sorption measurements and electron microscopy indicate small structure sizes with the redox active biobased polymer and carbon black particles bound together. No other synthetic additives or fluorinated binders were used, significantly increasing the sustainability of such electrodes. The electrodes showed a combination of faradaic and non-faradaic charge storage in the range of up to 80 mA h g^−1^ (referring to the total electrode mass), resulting from the quinone–hydroquinone redox couple in the biobased polymer and carbon black, respectively. Being free of any synthetic polymer, metal, or halogenated binder, these electrodes are not only more sustainable than other electrodes but they show also one of the highest capacities of biobased polymer electrodes reported so far.

## Conflicts of interest

There are no conflicts to declare.

## Supplementary Material

RA-009-C9RA00140A-s001
