# Supplementary material for: Vanillin decorated chitosan as electrode material for sustainable energy storage
Source: RSC Adv. 2019 Feb 6;9(8):4591–8. doi: 10.1039/c9ra00140a (PMC9060616; doi:10.1039/c9ra00140a)
Supplement: RA-009-C9RA00140A-s001 [file RA-009-C9RA00140A-s001.pdf]

Electronic Supplementary Material (ESI) for RSC Advances.
This journal is © The Royal Society of Chemistry 2019

Vanillin decorated chitosan as a binder-free electrode material for sustainable energy storage

*Ivan K. Ilic,[a] Maren Meurer,[a] Saowaluk Chaleawlert-umpon,[a,b] Markus Antonietti,[a] Clemens Liedel[a]**

[a] Department of Colloid Chemistry, Max Planck Institute of Colloids and Interfaces, Research Campus Golm, 14476 Potsdam, Germany

[b] National Nanotechnology Center, National Science and Technology Development Agency, Thailand Science Park, Pathumtani 12120, Thailand

*Clemens.Liedel@mpikg.mpg.de; Phone: +49 331 567 9552; Fax: +49 331 567 9502

Supporting Information

Synthesis procedures

Synthesis of ChiVan-0.5CB: Chitosan (0.5 g) was dissolved in 0.2 M acetic acid (35 mL) and stirred for 2 h. A solution of vanillin (0.48 g in 35 mL ethanol) was then added to the mixture. After 1 h of additional stirring at room temperature, $NaBH_3CN$ (0.77 g) was added and the reaction stirred further for 17 h. A precipitate formed which was dissolved by addition of glacial acetic acid (3 mL). After 1 h of further stirring, carbon black (0.5 g), that was previously grinded in an agate mortar, was added and stirred for 24 h. The mixture was neutralised using a 15% aqueous solution of Na_2CO_3 and stirred for 2 hours. The product was centrifuged by splitting the dispersion in two centrifugation tubes (max capacity 50 mL) and subsequently washed two times with water and two times with ethanol (each centrifugation was done at 4000 rpm for 3 minutes). The obtained solid was dried at 60 ^oC overnight.

Synthesis of ChiVan-2.5CB: Chitosan (0.5 g) was dissolved in 0.2 M acetic acid (35 mL) and stirred for 2 h. A solution of vanillin (0.48 g in 35 mL ethanol) was then added to the mixture. After 1 h of additional stirring at room temperature, $NaBH_3CN$ (0.77 g) was added and the reaction stirred further for 17 h. A precipitate formed which was dissolved by addition of glacial acetic acid (3 mL). After 1 h of further stirring, carbon black (2.5 g), that was previously grinded in an agate mortar, was added and stirred for 24 h. The mixture was neutralised using a 15% aqueous solution of Na_2CO_3 and stirred for 2 hours. The product was centrifuged by splitting the dispersion in two centrifugation tubes (max capacity 50 mL) and subsequently washed two times with water and two times with ethanol (each centrifugation was done at 4000 rpm for 3 minutes). The obtained solid was dried at 60 ^oC overnight.

Synthesis of chitosan-CB: Chitosan (0.5 g) was dissolved in 0.2 M acetic acid (35 mL) and stirred for 2 h. Ethanol (35 mL) was then added to the mixture. After 1 h of additional stirring at room temperature, $NaBH_3CN$ (0.77 g) was added and the reaction stirred further for 17 h. Glacial acetic acid (3 mL) was added. After 1 h of further stirring, carbon black (1.5 g), that was previously grinded in an agate mortar, was added and stirred for 24 h. The mixture was neutralised using a 15% aqueous solution of Na_2CO_3 and stirred for 2 hours. The product was centrifuged by splitting the dispersion in two centrifugation tubes (max capacity 50 mL) and subsequently washed two times with water and

two times with ethanol (each centrifugation was done at 4000 rpm for 3 minutes). The obtained solid was dried at 60 ^oC overnight.

Synthesis of chitosan-vanillin-CB: Chitosan (0.5 g) was dissolved in 0.2 M acetic acid (35 mL) and stirred for 2 h. A solution of vanillin (0.48 g in 35 mL ethanol) was then added to the mixture. The reaction was stirred further for 18 h. Glacial acetic acid (3 mL) was added. After 1 h of further stirring, carbon black (1.5 g), that was previously grinded in an agate mortar, was added and stirred for 24 h. The mixture was neutralised using a 15% aqueous solution of Na_2CO_3 and stirred for 2 hours. The product was centrifuged by splitting the dispersion in two centrifugation tubes (max capacity 50 mL) and subsequently washed two times with water and two times with ethanol (each centrifugation was done at 4000 rpm for 3 minutes). The obtained solid was dried at 60 ^oC overnight.

Synthesis of ChiVan: Chitosan (0.5 g) was dissolved in 0.2 M acetic acid (35 mL) and stirred for 2 h. A solution of vanillin (0.48 g in 35 mL ethanol) was then added to the mixture. After 1 h of additional stirring at room temperature, $NaBH_3CN$ (0.77 g) was added and the reaction stirred further for 17 h. A precipitate formed which was dissolved by addition of glacial acetic acid (3 mL). After 25 h of stirring the mixture was neutralised using a 15% aqueous solution of Na_2CO_3 and further stirred for 2 hours. The product was centrifuged by splitting the dispersion in two centrifugation tubes (max capacity 50 mL) and subsequently washed two times with water and two times with ethanol (each centrifugation was done at 4000 rpm for 3 minutes). The obtained solid was dried at 60 ^oC overnight.

Synthesis of ChiSyr: Chitosan (0.5 g) was dissolved in 0.2 M acetic acid (35 mL) and stirred for 2 h. A solution of Syringaldehyde (0.58 g in 35 mL ethanol) was then added to the mixture. After 1 h of additional stirring, at room temperature, $NaBH_3CN$ (0.77 g) was added and the reaction stirred further for 17 h. A precipitate formed which was dissolved by addition of glacial acetic acid (9 mL). After 1 h of further stirring, carbon black (1.5 g), that was previously grinded in an agate mortar, was added and stirred for 24 h. The mixture was neutralised using a 15% aqueous solution of Na_2CO_3 and stirred for 2 hours. The product was centrifuged by splitting the dispersion in two centrifugation tubes (max capacity 50 mL) and subsequently washed two times with water and two times with ethanol (each centrifugation was done at 4000 rpm for 3 minutes). The obtained solid was dried at 60 ^oC overnight.

Synthesis of ChiPhy: Chitosan (0.5 g) was dissolved in 0.02 M acetic acid (35 mL) and stirred for 2 h. A solution of p-hydroxybenzaldehyde (0.39 g in 35 mL ethanol) was then added to the mixture. After 1 h of additional stirring at room temperature, $NaBH_3CN$ (0.77 g) was added and the reaction stirred further for 18 h. Carbon black (1.5 g), that was previously grinded in an agate mortar, was added and stirred for 24 h. The mixture was neutralised using a 15% aqueous solution of Na_2CO_3 and stirred for 2 hours. The product was centrifuged by splitting the dispersion in two centrifugation tubes (max capacity 50 mL) and subsequently washed two times with water and two times with ethanol (each centrifugation was done at 4000 rpm for 3 minutes). The obtained solid was dried at 60 ^oC overnight.

Electrode preparation (Kraft lignin-CB): Kraft lignin (20 mg) and carbon black (40 mg) were grinded in a stainless steel jar using a planetary ball mill PM100 (Retsch). Propylene glycol (0.25 mL) and ethanol (0.75 mL) were used as solvent to prepare a processible slurry, which was further hand shaken and ultrasonicated before application. 40 µL of the obtained slurry was spread across a carbon paper (1.5 cm^2). The electrode was dried at 80 ^oC overnight.

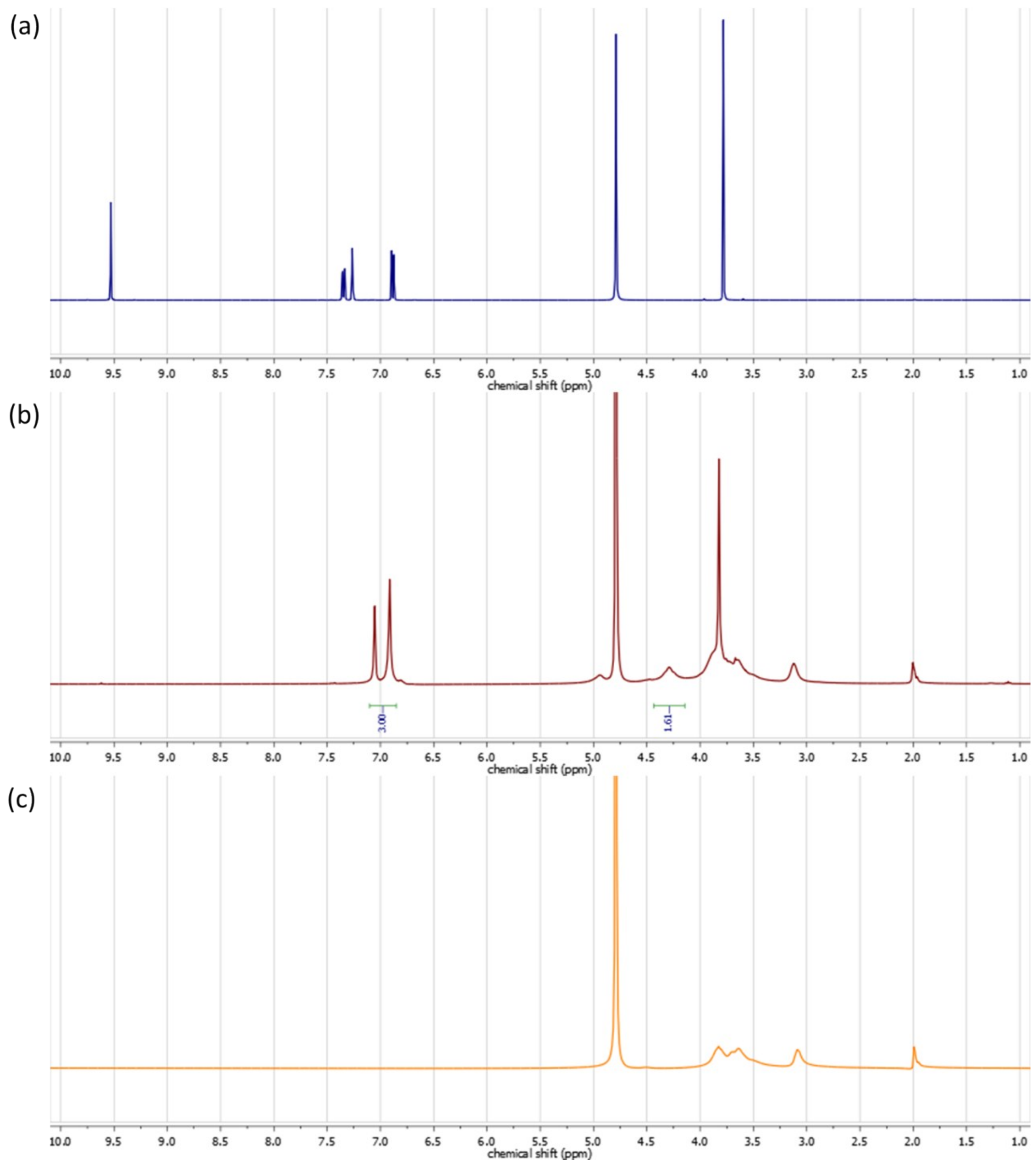

Figure S1. ^{1}H NMR spectra of (a) vanillin, (b) ChiVan, and (c) chitosan in 1% solution of deuterated acetic acid in deuterated water.

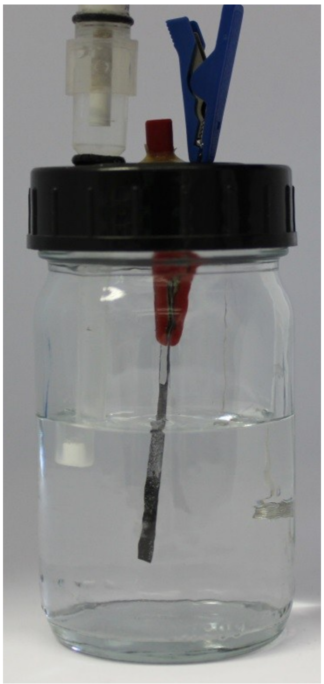

Figure S2. Cell used for electrochemical experiments.

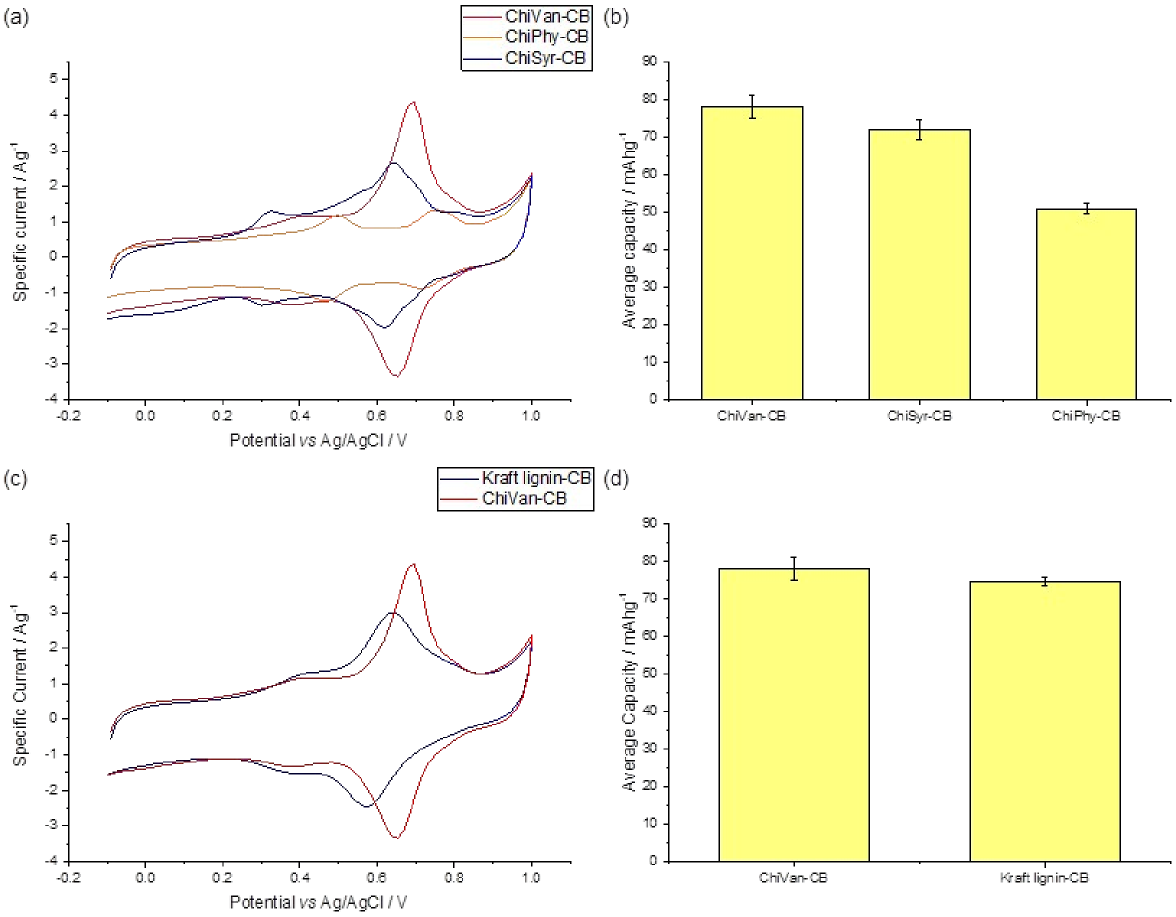

Figure S3. (a,c) Cyclic voltammetry and (b,d) average capacities calculated by integration of the cyclic voltammetry curves of (a,b) composites of carbon black with ChiVan, ChiSyr and ChiPhy and (c,d) composites of carbon black with ChiVan or Kraft lignin. All CV was tested in 1 M $HClO_4$ aqueous solution (5 mV s^{-1}).

By using chitosan based polymers bearing redox functionalities of lignin instead lignin itself it is possible to investigate redox properties of different functional groups in lignin, simply by exchanging vanillin as the redox-active group to other aldehydes. In this regard, we studied syringaldehyde (ChiSyr) and p-hydroxybenzaldehyde (ChiPhy) (Figure 4a,b). ChiSyr-CB shows similar CV curves to ChiVan-CB, however the orthoquinone redox couple is shifted to lower potential due to the electron donating effect of the additional methoxy group,[1] and the specific capacity is slightly lower. ChiPhy-CB shows no redox couple leading to lower specific capacity, as expected,[2] and the profile of the CV curve is similar to the one of chitosan-CB.

Additionally, the electrochemical performance of ChiVan was compared to the one of Kraft lignin (Figure 4c,d) by generating a composite of Kraft lignin and carbon black with the equivalent procedure as for ChiVan and a similar carbon black-to-redox active polymer ratio. Both CV profiles are similar, however with ChiVan’s pair of redox peaks being shifted to a higher potential, in fact one of the highest reported for organic electrodes.[3] Also the redox peaks of ChiVan are more clearly defined compared to the ones of Kraft lignin as ChiVan has only one redox active unit (guaiacyl groups), while Kraft lignin has both guaiacyl and syringyl units. Furthermore, ChiVan has a significantly higher specific capacity.

We note that guaiacyl rich lignins, those suitable for the production of vanillin, have proven to be ineffective for lignin-based cathode materials.[4] While the production of vanillin from lignin (and of chitosan-vanillin graft copolymers) requires additional synthesis steps and is consequently less economical than the usage of lignin itself, the good performance of ChiVan shows a way to improve charge storage in materials derived from guaiacyl rich lignins. One way to minimize the synthetic complexity and increase the sustainability of the electrodes would be to avoid the demanding separation steps of syringaldehyde from vanillin during vanillin synthesis and directly use a mixture of vanillin and syringaldehyde as obtained from lignin. The resulting capacity is expected to be similar to pure vanillin based electrodes.

Table S1. Combustive elemental analysis results of following samples.

	N [%]	C [%]	H [%]	O [%]
**Carbon black**	0.18	93.41	1.239	5.726
	0.15	93.71	1.014	5.556
**Avg**	0.165	93.56	1.1265	5.641
**ChiVan-0.40CB**	2.85	67.71	4.119	22.96
	2.83	67.38	4.226	22.921
**Avg**	2.84	67.545	4.1725	22.9405
**ChiVan-CB**	1.53	75.58	2.998	15.976
	1.57	76.35	3.207	17.453
**Avg**	1.55	75.965	3.1025	16.7145
**ChiVan-0.77CB**	1.14	77.8	2.691	15.96
	1.14	79.2	2.904	14.84
**Avg**	1.14	78.5	2.7975	15.4
**ChiVan**	4.84	52.72	6.148	35.485
	4.79	52.57	6.211	35.807
**Avg**	4.815	52.645	6.1795	35.646
**Chitosan-vanillin-CB**	1.56	76.26	2.706	18.023
	1.62	78.16	2.994	17.607
**Avg**	1.59	77.21	2.85	17.815
**ChiPhy-CB**	2.04	74.62	3.431	15.714
	2.91	64.4	3.993	16.371
**Avg**	2.475	69.51	3.712	16.0425
**ChiSyr-CB**	1.57	75.37	3.463	17.363
	1.43	73.86	3.048	17.363
**Avg**	1.5	74.615	3.2555	17.363
**chitosan-CB**	1.75	74.29	3.056	17.628
	1.76	75.58	3.07	17.49
**Avg**	1.755	74.935	3.063	17.559
**chitosan**	7.28	40.59	6.47	42.82
	7.47	41.27	6.705	43.943
**Avg**	7.375	40.93	6.5875	43.3815

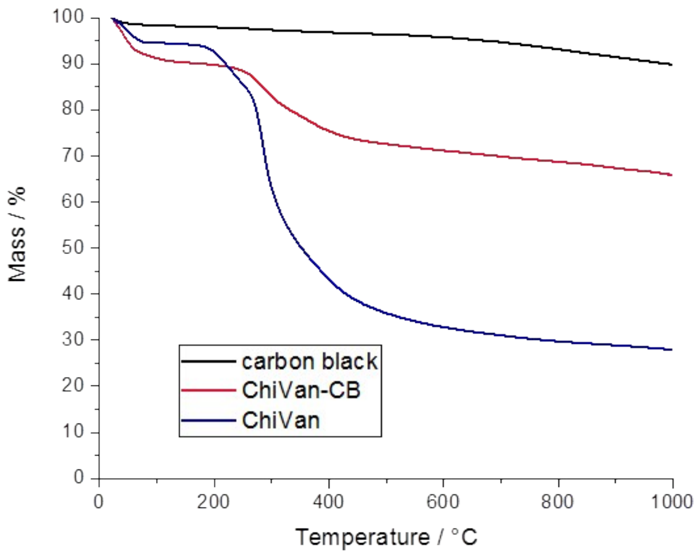

Figure S4. TGA of carbon black, ChiVan-CB, and ChiVan with a heating rate of 10 K min^{-1} under nitrogen atmosphere.

(a)

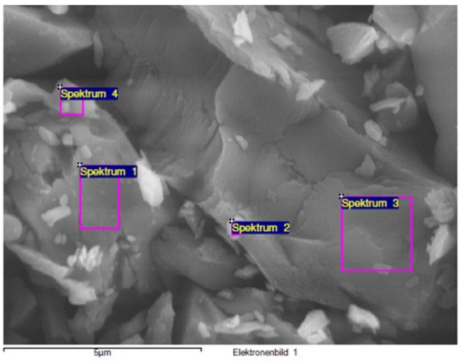

Processing option : All elements analysed (Normalised)

Spectrum	In stats.	C	N	O	Total
Spektrum 1	Yes	95.57	2.09	2.34	100.00
Spektrum 2	Yes	93.86	3.20	2.94	100.00
Spektrum 3	Yes	96.03	2.15	1.81	100.00
Spektrum 4	Yes	94.96	2.50	2.54	100.00
Mean		95.10	2.49	2.41	100.00
Std. deviation		0.94	0.51	0.47	
Max.		96.03	3.20	2.94	
Min.		93.86	2.09	1.81	

All results in weight%

(b)

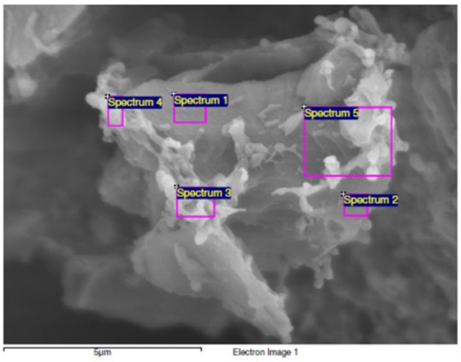

Processing option : All elements analysed (Normalised)

Spectrum	In stats.	C	N	O	Na	Total
Spectrum 1	Yes	92.09	3.74	3.87	0.31	100.00
Spectrum 2	Yes	93.54	2.91	3.19	0.36	100.00
Spectrum 3	Yes	85.72	7.35	6.45	0.48	100.00
Spectrum 4	Yes	85.51	6.16	7.66	0.67	100.00
Spectrum 5	Yes	89.71	4.59	5.28	0.42	100.00
Mean		89.31	4.95	5.29	0.45	100.00
Std. deviation		3.64	1.80	1.83	0.14	
Max.		93.54	7.35	7.66	0.67	
Min.		85.51	2.91	3.19	0.31	

All results in weight%

(c)

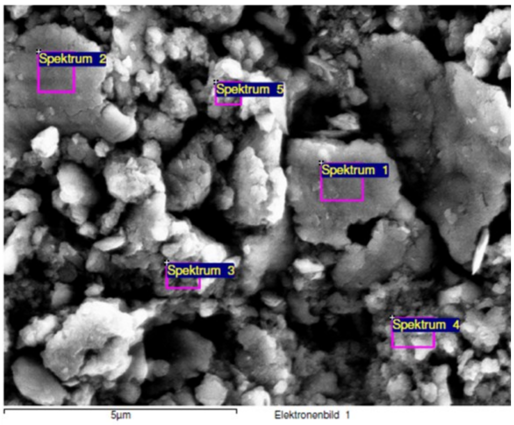

Processing option : All elements analysed (Normalised)

Spectrum	In stats.	C	N	O	Na	Total
Spektrum 1	Yes	87.83	4.64	6.97	0.56	100.00
Spektrum 2	Yes	86.78	5.15	7.60	0.46	100.00
Spektrum 3	Yes	88.55	4.65	6.32	0.48	100.00
Spektrum 4	Yes	85.95	5.93	7.50	0.61	100.00
Spektrum 5	Yes	87.84	5.05	6.65	0.46	100.00
Mean		87.39	5.08	7.01	0.52	100.00
Std. deviation		1.02	0.53	0.55	0.07	
Max.		88.55	5.93	7.60	0.61	
Min.		85.95	4.64	6.32	0.46	

All results in weight%

Figure S5. SEM images along with EDX measurements at different regions of (a) carbon black, (b,c) ChiVan-CB (b) before and (c) post-grind.

References

(1) Rębiś, T.; Nilsson, T. Y.; Inganäs, O. Hybrid Materials from Organic Electronic Conductors and Synthetic-Lignin Models for Charge Storage Applications. *J. Mater. Chem. A* **2016**, *4* (5), 1931–1940.

(2) Milczarek, Grzergorz; Inganas, O. Renewable Cathode Materials from Biopolymer/Conjugated Polymer Interpenetrating Networks. *Science* **2012**, *335*, 1468–1471.

(3) Song, Z.; Zhou, H. Towards Sustainable and Versatile Energy Storage Devices: An Overview of Organic Electrode Materials. *Energy Environ. Sci.* **2013**, *6* (8), 2280–2301.

(4) Admassie, S.; Nilsson, T. Y.; Inganäs, O. Charge Storage Properties of Biopolymer Electrodes with (Sub)tropical Lignins. *Phys. Chem. Chem. Phys.* **2014**, *16* (45), 24681–24684.
